# Impact of the HIV integrase genetic context on the phenotypic expression and *in vivo* dynamics of integrase inhibitor resistance mutations

**DOI:** 10.1186/1742-4690-10-S1-P56

**Published:** 2013-09-19

**Authors:** Nga Nguyen, Sylvie Rato, Francois Clavel, Constance Delaugerre, Fabrizio Mammano

**Affiliations:** 1 INSERM U941, Paris, France; 2 Hôpital Saint-Louis, Virology, Paris, France

## Background

HIV resistance to the integrase inhibitor raltegravir (RAL) in treated patients is characterized by three main distinct resistance pathways, for which the primary mutations are: N155H, Q148H/K/R, or Y143R/H/C. These genotypes may emerge sequentially, always carried by distinct viral genomes. The mechanism explaining the sequential emergence and *in vivo* dynamics along with viral escape from RAL treatment are poorly understood. We hypothesize that differences in the *in vivo* dynamics of HIV resistance to RAL, which must be a direct consequence of the phenotypic expression of viral resistance and/or of viral fitness, are due to the genetic context of the integrase carried by viral variants infecting individual patient.

## Materials and methods

We selected four patients infected with subtype-B HIV and for whom different prototypic evolutionary profiles of resistance to RAL were observed. Replication competent HIV molecular clones were constructed, which carried the integrase alleles from the dominant pre-therapy viral variants. Different primary resistance mutations were then introduced by site directed mutagenesis in the pre-therapy integrase sequences. We measured the impact of the genetic context of integrase on the phenotypic expression of resistance mutations by comparing baseline and mutated clones for viral fitness and resistance.

## Results

In 2 patients, mutation N155H emerged and persisted for several months (7 and 22, respectively) until treatment was changed. In 2 other patients Q148H emerged as the first detectable mutation or after a brief appearance of N155H.

Insertion of the mutation N155H had a moderate impact on virus fitness (residual fitness 40-60%) and induced a significant increase in resistance to RAL (15 to 60-fold) in all integrase contexts. In contrast, the impact of mutation Q148H differed substantially depending on the integrase allele in which it was inserted. In particular, it dramatically reduced the infectivity of one virus issued from a patient in whom this mutation did not emerge during RAL treatment. On the other hand, it preserved infectivity and it conferred high-level resistance in the context of the virus in which it rapidly emerged *in vivo.*

## Conclusions

Evolution toward different resistance genotypes is largely determined by the capacity of different integrase alleles present at baseline to minimize the effect of mutations on virus fitness, while allowing expression of resistance.

